# The First Cbk-Like Phage Infecting *Erythrobacter*, Representing a Novel Siphoviral Genus

**DOI:** 10.3389/fmicb.2022.861793

**Published:** 2022-05-10

**Authors:** Xuejing Li, Ruizhe Guo, Xiao Zou, Yanyan Yao, Longfei Lu

**Affiliations:** ^1^State Key Laboratory of Marine Environmental Science, Institute of Marine Microbes and Ecospheres, College of Ocean and Earth Sciences, Xiamen University (Xiang'an), Xiamen, China; ^2^Frontiers Science Center for Deep Ocean Multispheres and Earth System, College of Marine Life Sciences, Institute of Evolution and Marine Biodiversity, Ocean University of China, Qingdao, China; ^3^Qingdao Central Hospital, Qingdao, China; ^4^Weihai Changqing Ocean Science Technology Co., Ltd., Weihai, China

**Keywords:** *Erythrobacter*, Cbk-like phage, genomic analysis, phylogenetic analysis, novel genus

## Abstract

*Erythrobacter* is an important and widespread bacterial genus in the ocean. However, our knowledge about their phages is still rare. Here, a novel lytic phage vB_EliS-L02, infecting *Erythrobacter litoralis* DSM 8509, was isolated and purified from Sanggou Bay seawater, China. Morphological observation revealed that the phage belonged to Cbk-like siphovirus, with a long prolate head and a long tail. The host range test showed that phage vB_EliS-L02 could only infect a few strains of *Erythrobacter*, demonstrating its potential narrow-host range. The genome size of vB_EliS-L02 was 150,063 bp with a G+C content of 59.43%, encoding 231 putative open reading frames (ORFs), but only 47 were predicted to be functional domains. Fourteen auxiliary metabolic genes were identified, including phoH that may confer vB_EliS-L02 the advantage of regulating phosphate uptake and metabolism under a phosphate-limiting condition. Genomic and phylogenetic analyses indicated that vB_EliS-L02 was most closely related to the genus *Lacusarxvirus* with low similarity (shared genes < 30%, and average nucleotide sequence identity < 70%), distantly from other reported phages, and could be grouped into a novel viral genus cluster, in this study as *Eliscbkvirus*. Meanwhile, the genus *Eliscbkvirus* and *Lacusarxvirus* stand out from other siphoviral genera and could represent a novel subfamily within *Siphoviridae*, named *Dolichocephalovirinae*-II. Being a representative of an understudied viral group with manifold adaptations to the host, phage vB_EliS-L02 could improve our understanding of the virus–host interactions and provide reference information for viral metagenomic analysis in the ocean.

## Introduction

Viruses are the most abundant biological entities in the ocean (Suttle, 2005; Wigington et al., 2016). Bacteriophages, that is, viruses infecting bacteria, play important roles in the regulation of bacterial abundance, community structure, and carbon sequestration. Through viral lysis, bacterial cells can release dissolved organic carbon (DOC), some of which can be maintained in the ocean for the long term (Jiao et al., 2014; Zhang et al., 2018).

Aerobic anoxygenic photoheterotrophic bacteria (AAPB), which contain bacteriochlorophyll-a and lack light-harvesting complex II, are photoheterotrophic microorganisms and are wildly distributed in the epipelagic ocean (Kolber et al., 2001; Eiler, 2006; Jiao et al., 2007). Owing to its metabolic feature, AAPB could reduce the consumption of organic carbon, and play an essential role in the marine carbon cycle (Jiao et al., 2007, 2010; Zhang et al., 2016). To date, the isolated AAPB in the ocean mainly belongs to *Alphaproteobacteria* comprising the *Roseobacter* clade and Eryth-Citro clade, and *Gammaproteobacteria* (Béjà et al., 2002; Cho et al., 2007; Zheng et al., 2011, 2013). *Erythrobacter* was the first identified AAPB that is widely distributed in the ocean, especially in the eutrophic coastal waters and sediments (Shiba and Simidu, 1982; Yurkov et al., 1994; Nishimura et al., 2006; Lei et al., 2015). Genome and functional studies have shown that *Erythrobacter* has capacities for nitrate reduction, denitrification, aesculin hydrolysis, and multiple substrates utilization, such as amino acids, carbohydrates, and fatty acids (Koblížek et al., 2003; Wei et al., 2013; Zhuang et al., 2015; Fang et al., 2019). Furthermore, *Erythrobacter* is reported to have the capacity to degrade polycyclic aromatic hydrocarbons (Zhuang et al., 2015), which is considered a kind of refractory dissolved organic carbon (RDOC) (Chen et al., 2020), and may play an essential role in labile DOC (LDOC) acquisition for surrounding heterotrophs, especially in an oligotrophic environment (Zhang et al., 2016). Moreover, research showed that *Erythrobacter aquimaris* can produce poly-β-hydroxybutyrate (PHB), which is known as a material for degradable plastics and can be easily degraded by microorganisms in bioactive environments (Mostafa et al., 2020).

In consideration of the ecological significance of *Erythrobacter* in the ocean, our knowledge about the virus infecting *Erythrobacter* is limited. Currently, only one *Erythrobacter*-infecting phage vB_EliS-R6L was reported and is characterized by morphological icosahedral capsid and long tail, wide metabolic tolerance of temperature and pH, and ubiquitous distribution in the euphotic ocean (Lu et al., 2017). To gain a comprehensive understanding of the Erythrobacter phages, more research on their genomes and distribution patterns is required.

In the present study, a novel phage infecting *E. litoralis* DSM 8509, vB_EliS-L02, was isolated from nearshore seawater, and its morphological, physiological, genomic, phylogenetic, and ecological features were analyzed. Genomic features and evolutionary relationships indicated that phage vB_EliS-L02 was distant from the reported phages, could form a new viral cluster within the *Siphoviridae*, and represented a novel viral genus. Our study provides useful information for further analysis of the evolutionary and ecological roles of Erythrobacter phages in the ocean.

## Materials and Methods

### Bacterial Strains

*Erythrobacter litoralis* DSM 8509 was used as host bacteria for phage isolation. In addition to the strain mentioned above, 16 bacterial strains were used for host range analysis (Table 1). All bacterial strains were cultivated in an RO medium (containing 1 g/L yeast extract, 1 g/L tryptone, and 1 g/L sodium acetate, pH 7.5) at 30°C (Yurkov et al., 1999).

**Table 1:** Host range of the phage vB_EliS-L02.

**Strains**	**Best matched species (% Id of 16S rDNA)**	**Source and location**	Susceptibility to phage vB_EliS-L02^*^
*E. litoralis* DSM 8509		Cyanobacterial mat, Netherlands (Yurkov et al., 1994)	+
*Erythrobacter* JL 475		Surface sea water, South China Sea, China (Zheng et al., 2016)	+
*E. longus* DSM 6997		Seaweed *Enteromorpha linza*, Japan (Shiba and Simidu, 1982)	+
JL 917	*Erythrobacter citreus* RE35F/1 (99.72)	Surface sea water, Taiwan strait, China	–
JL 1267	*Erythrobacter* sp. MON004 (100.00)	Surface sea water, South China sea, China	–
JL 967	*Erythrobacter* sp. M71_W20 (100.00)	Surface sea water, Taiwan strait, China	–
JL 1833	*Erythrobacter flavus* BL16 (100.00)	Bottom sea water, South China sea, China	–
JL 1201	*Erythrobacter vulgaris* TVG01-C004 (99.80)	Surface sea water, West Pacific Ocean	–
JL 2316	*Erythrobacter* sp. CC-AMZ-30 L (97.12)	Surface sea water, Pacific Ocean	–
JL 658-2	*Erythrobacter citreus* RE35F/1 (99.66)	Surface sea water, Taiwan strait, China	–
JL 274-1	*Erythrobacter vulgaris* 022 2-10 (99.22)	Changjiang Estuary, China	–
*Roseobacter lineage* DFL12^T^		Cells of *Prorocentrum lima* (Swingley et al., 2007)	–
*Roseobacter lineage* DSM7001		Seaweed, Japan (Biebl et al., 2005)	–
*Citromicrobium* 354		Surface sea water, South China sea, China	–
*Citromicrobium* 3292	*Citromicrobium bathyomarinum* JF-1 (98.09)	Surface sea water, West Pacific Ocean	–
JL 2210	*Lutibacterium* sp. (100.00)	Surface sea water, Atlantic Ocean	–
JL 1614	*Halomonas* sp. (100.00)	Surface sea water, Pacific Ocean	–

* *, +, susceptible; –, resistant*.

### Phage Isolation and Purification

Seawater samples were collected by filtering through a 0.22-μm filter membrane (Millipore, USA) from Sanggou bay in Weihai, China, in October 2018. Double-agar layer method was used to examine the presence of phages (Pajunen et al., 2000). Every single plaque was collected, plaque-purified four times, and then cultured in sodium chloride–magnesium sulfate (SM) buffer (100 mM NaCl, 50 mM Tris, 10 mM MgSO_4_, and 0.01% gelatin, pH 7.5). Afterward, the isolated phages were stored at −80°C in SM buffer with several drops of chloroform.

### Phage Amplification and Transmission Electron Microscopy

The phage suspension was mixed with 1 L mid-log phase bacterial host culture and incubated at 30°C and 180 rpm in a shaker until fragmented and aggregated cells were observed. The phage particles were then harvested and purified following Zheng et al. (2018). Briefly, the phage–host culture medium was treated with DNase and RNase at the final concentration of 2 ng/L for 1 h at room temperature. Then NaCl concentration was adjusted to 1 M, and the lysates were incubated at 4°C for another 0.5 h. After centrifugation (8,000 × g), the supernatants were harvested and filtered through a 0.22-μm filter membrane (Millipore, USA). Then the filtrate was added with PEG 8000 (final concentration 10%, w/v), and placed at 4°C overnight. After centrifugation (10,000 × g, 45 min), the precipitate was resuspended in SM buffer and stored at 4°C overnight. Subsequently, the phages were purified using CsCl (gradient-density: 1.5 g/mL) gradient ultracentrifugation (Optima L-100 XP Ultracentrifugation, Beckman Coulter) at 200,000 × g, 4°C for 24 h. Then, CsCl was removed by dialysis against SM buffer.

Phage morphology was observed by using a transmission electron microscope (TEM). A 20 μl of purified phage suspension was deposited on a copper grid and negatively stained with 2% uranyl acetate for 10 min. Then the grid was loaded onto a 120 kV TEM (JEM-2100HC TEM, JEOL, Japan) for examination.

### One-Step Growth Experiments

One-step growth experiments were conducted according to previous studies (Pajunen et al., 2000; Wu et al., 2007). Briefly, after the bacterial strain *E. litoralis* DSM 8509 was grown to mid-log phase (OD600 nm 0.3–0.5), the isolated phage was added to the bacterial culture at a multiplicity of infection (MOI) of 0.1, and the mixture was kept at 30 °C for 10 min for phage adsorption. After centrifugation for 5 min at 8,000 × g, the supernatant was removed, and cell pellets were resuspended in RO medium and cultured at 30°C. At selected time points (0, 20, 40, 60, 80, 100, 120, 140, 160, 180, 200, 220 min, post-infection), the triplicate samples were subjected to serial dilutions separately, and the eclipse period was determined by double-layer plaque assay method (Pajunen et al., 2000). After incubation at 30°C for 24 h, plaques were enumerated, and titers were expressed as PFU/ml (plaque forming units per mL) for each plate.

### Host Range Determination

The host range of isolated phage was determined by spotting serial dilutions on double agar plates. Briefly, ~10^7^ PFU/ml phage suspension mixed with the potential host was added to each plate. Then the plates were incubated at 30°C for 24 h. The formation of phage plaques suggested the presence of lytic phages. A total of 17 marine bacterial strains were used for host range assessment, including 11 *Erythrobacter* strains, two *Roseobacter* strains, two *Citromicrobium* strains, one *Lutibacterium* strains, and one *Halomonas* strain.

### Phage Genome Extraction and Genome Sequencing

The CsCl-purified phage was first treated with DNase I (1 μg/ml) and RNase A (1 μg/ml) for 30 min at 37°C, and then treated with 100 μg/ml proteinase K and 10% SDS for 2 h at 56°C. Afterward, the genomic DNA was extracted twice with phenol:isoamyl alcohol (25:24:1), followed by two washes of chloroform:isoamyl alcohol (24:1). After centrifugation for 10 min at 12,000 rpm, the aqueous phage was precipitated with isopropanol (1:1 v/v) and sodium acetate (10:1 v/v) for 1 h at −20°C. The sample was then centrifuged at 12,000 rpm for 15 min, and the precipitated DNA was subsequently washed two times with 70% and 100% ethanol, dried, and resuspended in water.

The genome sequencing was performed on the Illumina Miseq platform with 2 × 250 bp paired-end reads, and CLC Genome Workbench software (43 × coverage) was used for genome assemblage.

### Bioinformatic and Phylogenetic Analyses

The putative open reading frames (ORFs) were identified using the GeneMarkS online server (http://exon.gatech.edu/Genemark/genemarks.cgi) (Besemer et al., 2001), Glimmer 3.0 (http://ccb.jhu.edu/software/glimmer/index.shtml) (Delcher et al., 2007), and ORF Finder online server (https://www.ncbi.nlm.nih.gov/orffinder/). Genes were BLAST searched against non-redundant (nr) protein database of NCBI for functional annotation (https://blast.ncbi.nlm.nih.gov/Blast.cgi), with a cut-off *E*-value of 10^−5^. The tRNA encoding genes were identified with tRNAscan-SE 2.0 online server (http://lowelab.ucsc.edu/tRNAscan-SE/) (Lowe and Chan, 2016; Chan and Lowe, 2019).

To determine the viral taxonomic family of the isolated phage and its host taxonomic group, the phylogenetic tree was constructed using the Viral Proteomic Tree server (VipTree, https://www.genome.jp/viptree/) based on the genome-wide sequence similarities computed by tBLASTx (Nishimura et al., 2017).

To investigate the taxonomy position of phage vB_EliS-L02 in the siphovirus, phylogenetic analysis was conducted with Virus Classification and Tree Building Online Resource (VICTOR; https://ggdc.dsmz.de/victor.php) (Meier-Kolthoff and Göker, 2017) using the Genome-BLAST Distance Phylogeny (GBDP) method under settings recommended for prokaryotic viruses (Meier-Kolthoff et al., 2013). The phylogenetic tree was visualized by iTOL (https://itol.embl.de) (Letunic and Bork, 2021). Taxon boundaries at the genus and subfamily level were estimated with the OPTSIL program (Göker et al., 2009) with the recommended clustering thresholds (Meier-Kolthoff and Göker, 2017) and an F value (fraction of links required for cluster fusion) of 0.5 (Meier-Kolthoff et al., 2013).

Average nucleotide identity by orthology (OrthoANI) was obtained with OrthoFinder by using all-*vs*-all BLASTp analysis to determine the genomic similarity between two genomes (Emms and Kelly, 2015, 2019). vConTACT 2.0 was used to calculate the similarity scores between every pair of genomes selected in this study (Teeling et al., 2004; Bolduc et al., 2017). All the taxonomic information of the phages was expanded using BLASTp with a cut-off *E*-value of 10^−5^ and covering an alignment region of more than 50%.

For the maximum-likelihood phylogenetic trees of major capsid (ORF 9) and portal protein (ORF 6) construction, orthologs were obtained by BLASTp with nr protein database in NCBI, sequences alignments were constructed with Clustal W integrated with MEGA X, and run with MEGA X using “LG+G” model with 1,000 bootstrap replications (Kumar et al., 2018). For phoH (ORF 2) phylogenetic tree construction, reference amino acid sequences from viruses and hosts were gathered from NCBI GenBank and aligned using MUSCLE (Edgar, 2004). Then the phylogenetic tree was constructed using IQtree 2.0 with a bootstrap of 1,000 after evaluating the optimal amino acid substitution models (Nguyen et al., 2015), and visualized using iTOL (https://itol.embl.de) (Letunic and Bork, 2021).

### Genome Recruitment

To evaluate the distribution of the phage vB_EliS-L02 in different environments, the ORF amino acid sequences were used for homologs recruitment from the Pacific Ocean Virome (POV) database. The reads were recruited by tBLASTn with a cut-off of *E* ≤ 10^−5^, an alignment value ≥ 30, and a score value ≥30, as previously described (Lu et al., 2017). Then the genome of the isolated phage was mapped to the Global Ocean Viromes 2.0 (GOV 2.0) (Gregory et al., 2019) using minimap2 (2.17–r941) (Li, 2018). Reads Per Kilobase per Million mapped reads (RPKM) was used for comparing the relative abundance between different viruses (Mortazavi et al., 2008), and the depth of coverage was calculated using CoverM v0.6.0 (https://github.com/wwood/CoverM) with the following parameters: -m rpkm–min-read-percent-identity 0.95–min-read-aligned-percent 0.75) (Li et al., 2021).

### Nucleotide Sequence Accession Number

The nucleotide sequence of the phage vB_EliS-L02 was submitted to the GenBank database under access no. OL955261.

## Results and Discussion

### Isolation and Basic Characterization

**Figure 1:**
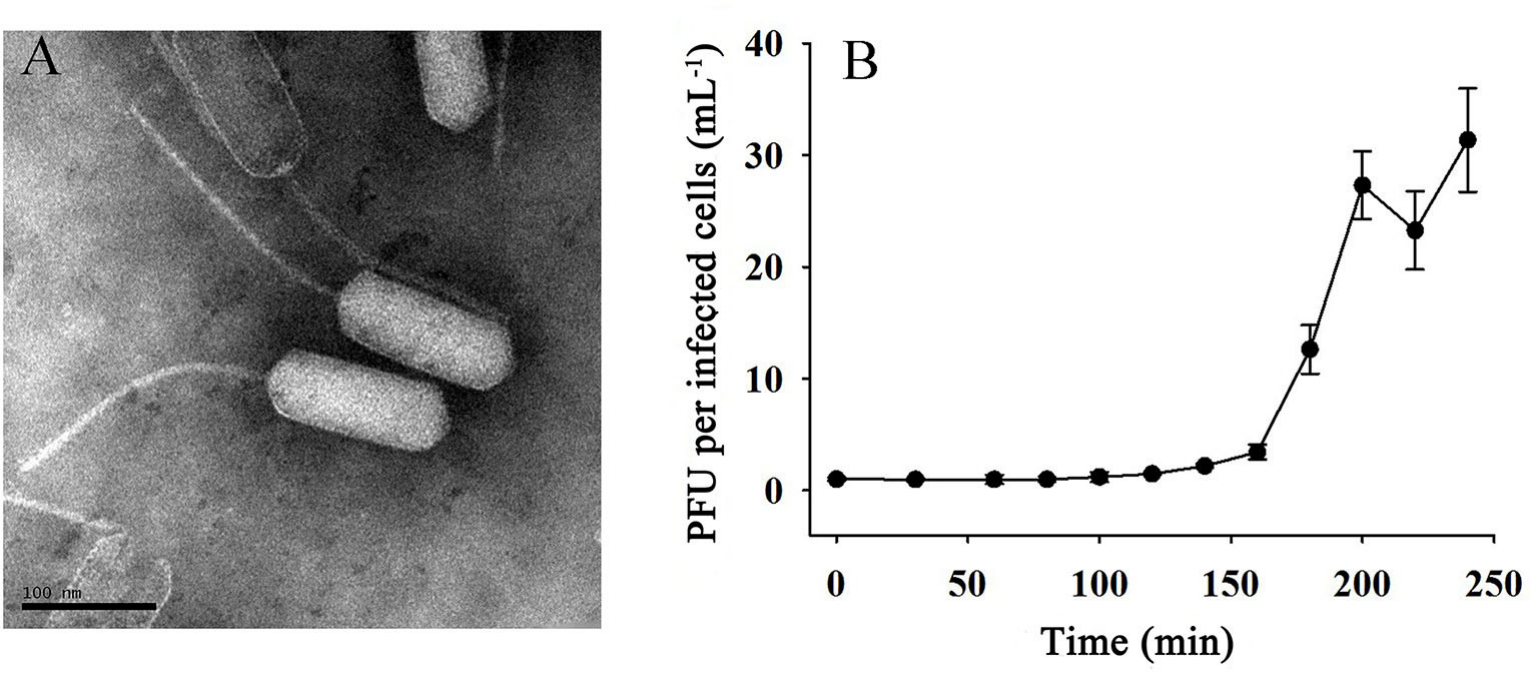
**(A)** Transmission electron microscopy (TEM; scale bar 100 nm) image and **(B)** one-step growth curve of phage vB_EliS-L02.

Phage vB_EliS-L02 was isolated from surface water in Sanggo bay in October 2018 using the double-agar layer method and found to form small, clear, and round plaques on the lawn of *Erythrobacter*. TEM analysis revealed that phage vB_EliS-L02 was a siphovirus, the diameter of the long prolate head was 159.4 ± 4.4 nm, and the length of the tail was 240.0 ± 5.1 nm (Figure 1A). The morphology of phage vB_EliS-L02 was remarkably distinct from the only reported Erythrobacter phage vB_EliS-R6L (Lu et al., 2017), and it is similar to a few Cbk-like phages, namely, Roseobacter phage DSS3P8 (Zhan et al., 2016), Phaeobacter phage MD18 (Urtecho et al., 2020), Caulobacter phiCbk-like phage (Gill et al., 2012), and Sphingobium phage Lacusarx (Nielsen et al., 2017).

The one-step growth curve of phage vB_EliS-L02 showed that the latent period lasted for ~150 min (Figure 1B). Then a rapid increase of PFU lasted from 150 to 200 min, with the burst size reaching ~45 PFU per infected cell. Chloroform sensitivity tests showed that phage vB_EliS-L02 was insensitive to chloroform, indicating that the phage was most likely unenveloped.

To determine the host range of phage vB_EliS-L02, 17 bacterial strains belonging to the genera *Erythrobacter, Roseobacter, Citromicrobium, Lutibacterium*, and *Halomonas* were used. Phage vB_EliS-L02 could only infect *E. litoralis* DSM 8509, *Erythrobacter* JL 475, and *E. longus* DSM 6997, suggesting a relatively narrow host range (Table 1). The phylogenetic analysis of *Erythrobacter* strains showed a close evolutionary relationship between the three strains that could be infected by phage vB_EliS-L02 as mentioned above (Zheng et al., 2016), suggesting that phage vB_EliS-L02 may only prefer to infect a specific *Erythrobacter* clade.

### Genomic Features

Phage vB_EliS-L02 contained a 150,063-bp double-stranded linear DNA genome. The G + C content was 59.43%, lower than the 65.2% of its host *Erythrobacter* (GenBank accession no. NZ_CP017057) and 66.5% of the other Erythrobacter phage vB_EliS-R6L (Lu et al., 2017). The genomic features of phage vB_EliS-L02 were then compared with the other 11 Cbk-like phages and showed that the most closely related phage was Sphingobium phage Lacusarx (Nielsen et al., 2017; Table 2).

**Table 2:** Genomic features of phage vB_EliS-L02, phage vB_EliS-R6L, and other Cbk-like *siphoviridae*.

**Phage name**	**Size (Kb)**	**GC%**	**tRNA**
vB_EliS-L02	150.063	59.43	29
vB_EliS-R6L	65,675	66.50	0
Lacusarx	130.138	60.20	24
DSS3P8	146.135	56.30	24
MD18	149.262	58.30	32
phiCbK	215.71	66.20	32
CcrSwift	219.216	66.10	27
CcrRogue	223.72	66.10	23
CcrBL10	220.934	65.70	26
CcrColossus	279.967	62.20	28
CcrPW	308.141	62.20	29
CcrSC	317.489	64.20	39
CcrBL9	322.272	63.70	37

Twenty-nine tRNA (20 unique) were identified in the genome of phage vB_EliS-L02. A high number of tRNAs were detected in the genomes of other jumbo phages, such as Cbk-like and T4-like phages (Skliros et al., 2016; Yang et al., 2021). These intrigued tRNAs might relate to phage integration in the prokaryotic chromosomes and usage of codons, which could lead to higher translational efficiency of phage genes than that of their hosts (Bailly-Bechet et al., 2007; Delesalle et al., 2016), especially at the late stages of infection when the host rRNA pool might be degraded (Yang et al., 2021). Moreover, the carried tRNAs in jumbo phages could prolong the replication period amid host cell shutdown, and reduce the selection pressure under the long infection period (Yang et al., 2021).

Functional prediction of phage vB_EliS-L02 genome showed 231 ORFs, accounting for 63.6% of the total genome (Supplementary Table 1). Each identified ORF was characterized using BLAST analysis in the NCBI database, 116 ORFs were predicted as functional ORFs, and 50 ORFs were annotated as functional domain categories. Further analysis showed that four functional modules were classified within the functional ORFs, including phage packing, phage structure protein, host lysis, and DNA replication, recombination, and repair functions (Supplementary Table 2). In addition, ORFs 21, 108, and 148 were predicted to encode DUF2460, DUF2958, and DUF1508 domain-containing proteins, respectively, with unknown functions (Supplementary Table 2).

Four genes were associated with phage packing, including portal protein (ORF 6) and tail tape measure protein (ORF 20), which were located at the anterior of the genome, and terminase small subunit (TerS) (ORF 189) and terminase large subunit (TerL) (ORF 190), which were located at the posterior of the genome. Portal protein plays essential roles in phage packing, such as procapsid assembly, screening mutations during packing, tail assembly, and DNA passaging into the capsid (Isidro et al., 2004). Tail tape measure protein dictates the tail length of the tailed bacteriophages and could facilitate DNA transit to the cell cytoplasm during infection (Mahony et al., 2016). The terminase encoded by phage comprises two subunits, TerS and TerL, and could initiate head packaging and process viral DNA into procapsids (Feiss and Rao, 2012).

Eight phage structure–related genes were predicted in the genome, located at the anterior position of the genome. Two genes (ORFs 8 and 13) were predicted to encode virions and phage structure proteins. Major capsid protein (ORF 9) and minor capsid protein (ORF 11) were identified in the genome; the former is the major structural component of icosahedral virus particles, and the latter is believed to enhance the stability of capsid structure by forming protein–protein interactions (Thammatinna et al., 2020). Four ORFs were related to tube structure, including major tail tube protein (ORF 17), which is used for viral genome delivery to the host cells (Davidson et al., 2012); tail fiber protein (ORF 25), which is responsible for host-range determination (Garcia-Doval and van Raaij, 2012); and two tail proteins (ORFs 24 and 26).

Five genes were involved to host lysis, of which, two genes were related to host adhesion, including ORFs 22 and 27 that are contained in F5/8 type C domain and LamG domain, respectively. These two domains were widely distributed in jumbo phages and host cells that play roles in adhesion (Iyer et al., 2021). ORF 23 (NlpC/P60 family protein) and ORF 28 (1,6-anhydro-*N*-acetylmuramyl-L-alanine amidase, AmpD) are related to host cell wall lysis, in which NlpC/P60 could degrade the peptidoglycan and destabilize the cell wall of hosts (Anantharaman and Aravind, 2003), and AmpD could cleave the cell wall peptidoglycan as an endolysin (Catalao et al., 2013). In addition, ORF 74 was predicted to be rIIA lysis inhibitor that can delay lysis when sensing externally related phage attacking the host (Abedon, 2019).

A total of 16 genes were related to DNA replication, recombination, and repair functions. Seven genes participated in phage DNA replication, recombination, and repair functions directly, and most of them were located in the upstream region of the phage vB_EliS-L02 genome. Two types of DNA polymerases were predicted, including PolC-type DNA polymerase III (ORF 55) and thermostable DNA polymerase I (ORF 60). ORFs 40 (RNA polymerase alpha subunit C-terminal domain protein) and 72 (RNA polymerase-associated protein) are contained in a DNA-dependent RNA polymerase that can help establish the virus at the early stage after infection (Ahn et al., 1994; Iyer et al., 2021). Three ORFs were related to DNA assembly, identified as ribonucleoside-diphosphate reductase (NrdA) subunit beta (ORFs 36 and 37) and subunit alpha (ORF 38), which are tightly coupled to DNA biosynthesis. NrdA catalyzes the first committed step in the biosynthesis of the deoxyribonucleoside triphosphates and could be allosteric effectors that regulate the DNA replication of the phage (Brown and Reichard, 1969; Tseng et al., 1988). Nine ORFs were involved in the regulation of DNA replicating, recombining, and repairing. ORF 32 encodes a DNA ligase that could participate in the processes of DNA replication, recombination, and repair. ORF35 encodes an HTH-type transcriptional regulator, which is usually found to be a DNA-binding domain in the transcriptional regulator, and associated with regulatory metabolism in gene expression (Yu et al., 2019). ORF 47, codifying for an ATP-dependent RecD-like DNA helicase, is implicated in DNA repair (Köppen et al., 1995). ORF 73 encodes a transposase, which may relate to the regulation of gene expression. ORF 77 encodes tyrosine recombinase that mediates unidirectional site-specific recombination between two DNA recognition sequences by mediating strand cleavage through catalyzing tyrosine (Groth and Calos, 2004). Other than that, tyrosine recombinases could protect genome integrity in phages by promoting post-replicative segregation of plasmids and circular chromosomes during cell division, and drive the movement of mobile genetic elements which can promote the phages' integration and transfer in bacterial genomes (Smyshlyaev et al., 2021). ORF188 encodes crossover junction endodeoxyribonuclease (RusA), which is involved in the viral processes of DNA replication, recombination, and repair (Mandal et al., 1993). Two AAA family ATPase-encoded genes (ORF 85 and 117) were predicted, which should participate in DNA replication and recombination (M Iyer et al., 2021). In addition, a tRNA repair-related ORF (ORF 85) was identified in phage vB_EliS-L02 genome, encoding RNA-splicing ligase that participates in tRNA supplementary by ligating cleaved tRNAs (Burroughs and Aravind, 2016).

### Auxiliary Metabolic Genes in the Genome of Phage vB_EliS-L02

Since the development of genome sequencing techniques, phage genomes were found to acquire some ecologically important genes from their hosts or other phages by horizontal gene transfer (Lawrence et al., 2002). These genes are usually referred to as auxiliary metabolic genes (AMGs), which are thought to increase viral replication (Hurwitz and U'Ren, 2016) and affect the metabolisms of hosts (Thompson et al., 2011). In this study, 14 ORFs were predicted to be AMGs within the phage vB_EliS-L02 genome.

ORF 2 encodes *phoH* gene, which belonged to a Pho regulon, and is speculated to play a role in regulating the phosphate uptake and metabolism under a phosphate-limiting condition (Wanner, 1996; Hsieh and Wanner, 2010; Luo et al., 2020). Phosphorus, which serves as a major element for nucleotide biosynthesis, is always a limiting factor in the ocean, especially for oligotrophic waters (Paytan and McLaughlin, 2007). Thus, proteins related to phosphorus acquisition such as phoH, pstS, and phoA, which may assist in phosphorus acquisition during viral infection (Hsieh and Wanner, 2010), are ubiquitous in microorganisms in the ocean. *phoH* has been used as a marker gene for phage diversity analysis, and the result showed a wide distribution pattern and universal existence across prokaryotes and phages (Goldsmith et al., 2011). In our study, phoH was also used as a marker protein to investigate evolution relationships.

Four ORFs were affiliated with pyrimidine metabolism. ORF 31 encodes FAD-dependent thymidylate synthase (ThyX), which could catalyze dUMP to form dTMP in the presence of reduced nucleotides and oxidized flavin adenine dinucleotide (FAD). The Thy–dUMP complexes with bound FAD show high efficient NAD(P)H oxidase activity and can affect DNA metabolism (Graziani et al., 2004). ORF 44 encodes nucleotide pyrophosphohydrolase (MazG). MazG homologs are implicated as programmed cell death regulators *via* reducing the levels of the central alarmone molecule (p)ppGpp in *Escherichia coli* (Gross et al., 2006), while preferring dGTP and dCTP as its substrates and play a role in deoxyribonucleotides hydrolyzing in the phage (Rihtman et al., 2019). ORF 46 encodes deoxynucleotide monophosphate kinase (dNMP kinase), which is an enzyme used for the rapid synthesis of the DNA for phage, catalyzing the phosphorylation of deoxynucleoside monophosphates to the respective diphosphates (Mikoulinskaia et al., 2003). ORF 69 contains a cytidine and deoxycytidylate deaminase zinc-binding region. Deoxycytidylate deaminase (dCMP deaminase) catalyzes the deamination of deoxycytidine 5'-monophosphate nucleotide (dCMP) to uridylate (dUMP), providing the major flux of dUMP production (Maley and Maley, 1990).

Two ORFs were linked to nicotinate and nicotinamide metabolism, including ORF 102 (Nicotinate phosphoribosyltransferase) and ORF 103 (bifunctional NMN adenylyltransferase/nudix hydrolase). These two enzymes belonged to pyridine nucleotide adenylyltransferase (PNAT) family and catalyze the biosynthesis of nicotinamide adenine dinucleotide (NAD^+^) (Singh et al., 2002), an essential cofactor for innumerable metabolic processes. This NAD^+^ biosynthesis belongs to the NAD^+^ salvage pathway, which could be found in large phage genomes (Miller et al., 2003; Yamada et al., 2010; Lee et al., 2017), and provided more substrates for DNA replication and metabolic regulation (Lee et al., 2017).

Moreover, other AMGs involving metabolic regulation were detected in the phage vB_EliS-L02 genome, which provides relaxed pathways for viral metabolism. ORF 34 encodes a 5'-nucleotidase that catalyzes the hydrolysis of phosphate esterified at carbon 5' of the ribose and deoxyribose portions of nucleotide molecules (Zimmermann, 1992). ORF 83 encodes a proteasome, which plays an important role in the regulation of protein degradation, cell cycle progression, DNA repair, apoptosis, and receptor signaling in host cells, and may enhance the efficiency of viral infection (Ros and Kempf, 2004; Volcy and Dewhurst, 2009). ORF 92 encodes a metallophosphoesterase that belongs to phosphatases and could participate in various regulatory metabolisms including mediating the dephosphorylation of certain proteins and phosphodiester bonds to regulate viral transcription (Cohen and Cohen, 1989). Peptidyl-tRNA hydrolase (ORF 109) is identified in the genome, which is broadly distributed in nature. Peptidyl-tRNA hydrolase can rapidly clear peptidyl-tRNA that may lead to cell death with accumulation by cleaving the ester bond between the peptide and tRNA, and plays a critical role in protein biosynthesis in the host cells (Das and Varshney, 2006; Sharma et al., 2014). ORF 130 belonged to the nucleotidyltransferase domain that participates in the repair of the 3′ terminus of defective tRNA molecules in cells (Shan et al., 2008). ORF 134 encodes thioredoxin (TRX), which is known as a hydrogen donor for ribonucleotide reductase (Sengupta and Holmgren, 2014). ORF 166 was predicted to be membrane protease (FtsH), which participates in stress response and protein quality control, and regulates the circuits of membrane proteins regulation in host cells (Langklotz et al., 2012). Otherwise, FtsH is related to the late host lysis step of the phage (Roces et al., 2013).

### Phylogenetic and Comparative Genomic Analyses

**Figure 2:**
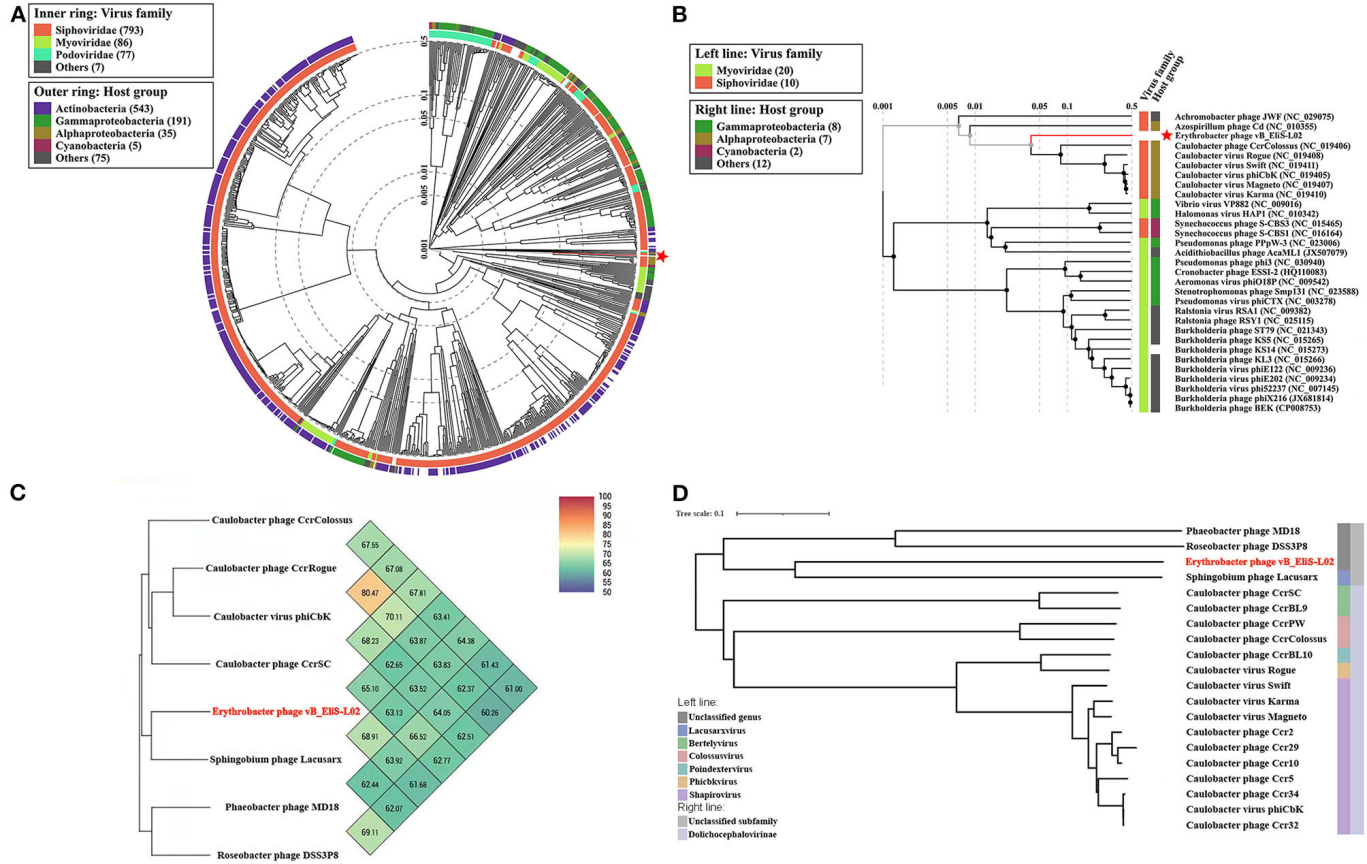
Phylogenetic and comparative analysis of phage vB_EliS-L02. **(A)** Determination of taxa and host group by a proteomic tree using VipTree including all compared sequences. The colored rings represent for virus family (inner ring) and host group (outer ring). **(B)** Phylogenetic tree including vB_EliS-L02 and 30 closest virus genomes. The branch length scale was calculated as log values. The left and right color bars indicate the taxonomic virus family and host group, respectively. The red star marks the position of vB_EliS-L02. **(C)** Heat map showing OrthoANI values of vB_EliS-L02 and the most genetically similar phages. The values were calculated by using OAT software. **(D)** Whole genome–based phylogenetic tree constructed by VICTOR with phage vB_EliS-L02 and other Cbk-like phages. Each genus is represented by a unique color.

To gain a better understanding of the evolutionary dynamics of phage vB_EliS-L02, the phylogenetic tree was constructed with genome sequences by using the Viral Proteomic Tree server (VipTree) (Figures 2A,B). Phage vB_EliS-L02 was clustered with the Caulobacter viruses that belonged to Cbk-like phage within the family *Siphoviridae*, and this result consisted of the morphological analysis. Subsequently, we analyzed our whole genome sequence using BLASTn search on GenBank, and the results showed a low query coverage (<7%), suggesting that phage vB_EliS-L02 is a new phage member compared with the published phage genomes. Then, we compared the similarity of phage vB_EliS-L02 and other 11 Cbk-like phages (Figure 2C). The results showed that the most similar phage was Sphingobium phage Lacusarx, which belonged to the genus *Lacusarxvirus* within the family *Siphoviridae*, with an average nucleotide identity (ANI) value of 68.91%. This ANI value was lower than the mechanistic demarcation criterion (70%) for phage genera (Turner et al., 2021). To determine the taxonomic position of phage vB_EliS-L02 in Cbk-like phages, whole genome–based phylogenetic analysis was conducted for phage vB_EliS-L02 and other 19 Cbk-like phages (Figure 2D). The results showed that phage vB_EliS-L02 was clustered with Sphingobium phage Lacusarx and was distant from subfamily *Dolichocephalovirinae*. Integrating with OrthoANI analysis, phage vB_EliS-L02 should be classified as a new viral genus, named *Eliscbkvirus*, and could form a subfamily with the genus *Lacusarxvirus*, named *Dolichocephalovirinae*-II.

**Figure 3:**
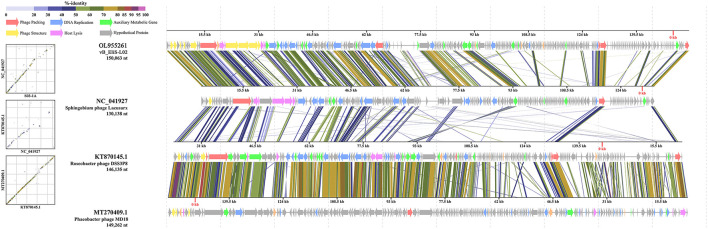
Genome comparisons between phage vB_EliS-L02 and typical Cbk-like phages. The ORFs are represented by arrows. The predicted functional domains represented arrows in different colors. The shading below each genome indicates sequence similarities between the genomes.

Then, comparative genomic analysis was performed between phage vB_EliS-L02 and typical Cbk-like phages, including Sphingobium phage Lacusarx (GenBank accession number NC_041927), Roseobacter phage DSS3P8 (KT870145), and Phaeobacter phage MD18(MT270409) by BLASTx. Phage vB_EliS-L02 genome showed relatively higher similarity with Sphingobium phage Lacusarx and was significantly different from other Cbk-like phage genomes (Figure 3). The shared ORFs between phage vB_EliS-L02 and phage Lacusarx was 27.3%, including 23 functional domains, with identities ranging from 35.5 to 79.2%. And only 16 genes were shared by phage vB_EliS-L02 and phage Lacusarx with identities higher than 50%, accounting for 15.5% of the total length of phage vB_EliS-L02 genome, including one structure protein (major capsid protein, the amino acid identity of 79.2%, calculated by BLASTp), one phage packaging–related protein (terminase-like family protein, 66.6%), one host lysing–related protein (F5/8 type C domain protein, 51.7%), 11 DNA replication related proteins (52.0–70.3%), and two nicotinate and nicotinamide metabolism–related proteins (51.0–64.8%). Considering only a few ORFs with higher identities were found between phage vB_EliS-L02 and the closed related phage, the result verified that phage vB_EliS-L02 represents a new phage genus.

**Figure 4:**
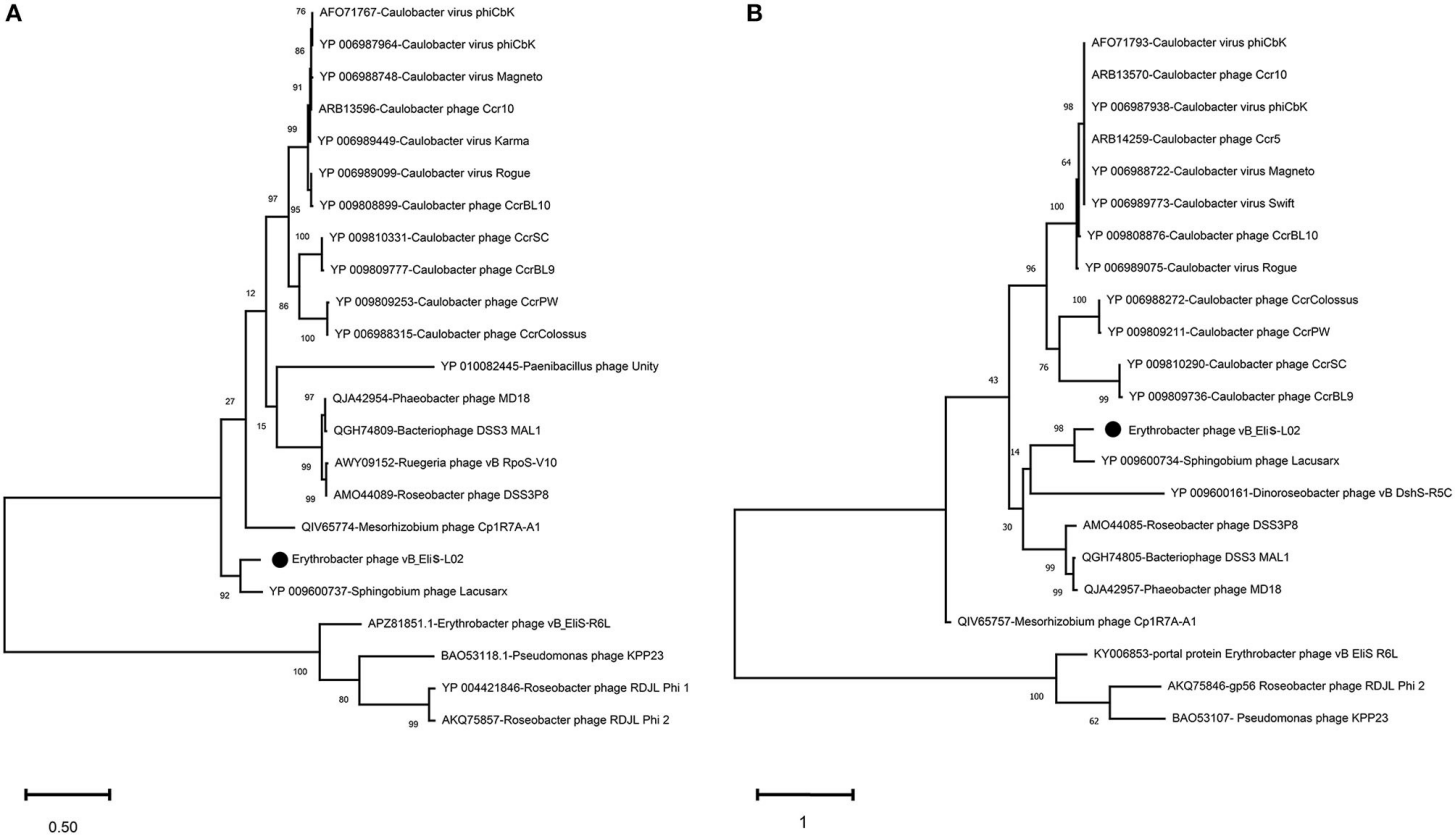
Maximum-likelihood phylogenetic trees based on **(A)** major capsid protein and **(B)** portal protein.

**Figure 5:**
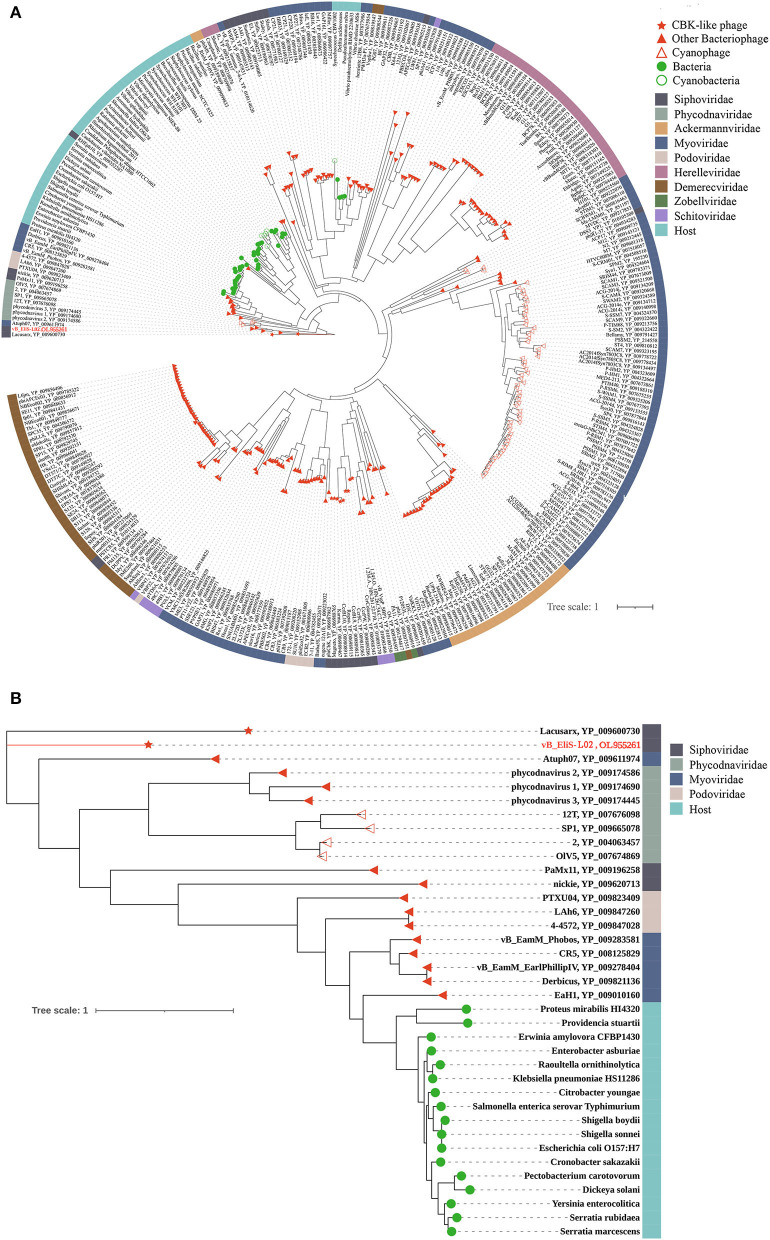
**(A)** phoH-based phylogenetic tree constructed by IQtree showing the relationships among phages and their hosts and **(B)** local phylogenetic tree showing the details of the distribution patterns related to phage vB_EliS-L02. Each virtual family or bacteria is represented by a unique color. Phage vB_EliS-L02 is colored in red.

To further validate the novelty of phage vB_EliS-L02, the phylogenetic trees were constructed by using major capsid protein (MCP), portal protein (Figures 4A,B), and phoH (Figure 5). The former two are ubiquitous and conserved in phage genomes, and are usually used as gene markers for phylogenetic analysis, while the latter one is widely distributed in viruses and hosts, and has been proved to be an effective signature gene for phage diversity analysis in many environments (Goldsmith et al., 2011; Wang et al., 2016; Li et al., 2019). Results of three phylogenetic trees all confirmed that phage vB_EliS-L02 showed a long evolutionary distance from other known phages, and represented a very new virus taxon. Results of phylogenetic trees based on MCP and portal protein showed that Sphingobium phage Lacusarx was the only phage clustered together with phage vB_EliS-L02. And, these two phages were clustered with some Roseobacter phages according to the phylogenetic tree of portal protein, suggesting a relatively close evolutionary distance among them (Zhan et al., 2016; Urtecho et al., 2020). Moreover, the evolutionary relationships, based on phoH among vB_EliS-R6L, other viruses, and their hosts, revealed that phage vB_EliS-L02 formed a distinctly separate cluster, with the closest relationship with Sphingobium phage Lacusarx. And the phylogenetic tree of phoH also showed that these two phages had far genetic distances from other isolated phages and bacteria.

### Environmental Distribution Pattern

Through metagenome recruit, a total of 4,834 reads within 49 ORFs were obtained. The results showed that the homologs were distributed in diverse ocean environments, including coastal waters, open oceans, and the intermediate waters between them, and most commonly detected in the coastal waters (76.6%), followed by open oceans (13.4%) and intermediate waters (10.0%). Further analysis showed a similar distribution pattern of phage vB_EliS-L02 homologs by using ORF 9 (Major capsid protein), ORF 6 (Portal protein), and ORF 2 (phoH). This distribution profile is consistent with the host *Erythrobacter* (Shiba and Simidu, 1982; Jiao et al., 2007).

**Figure 6:**
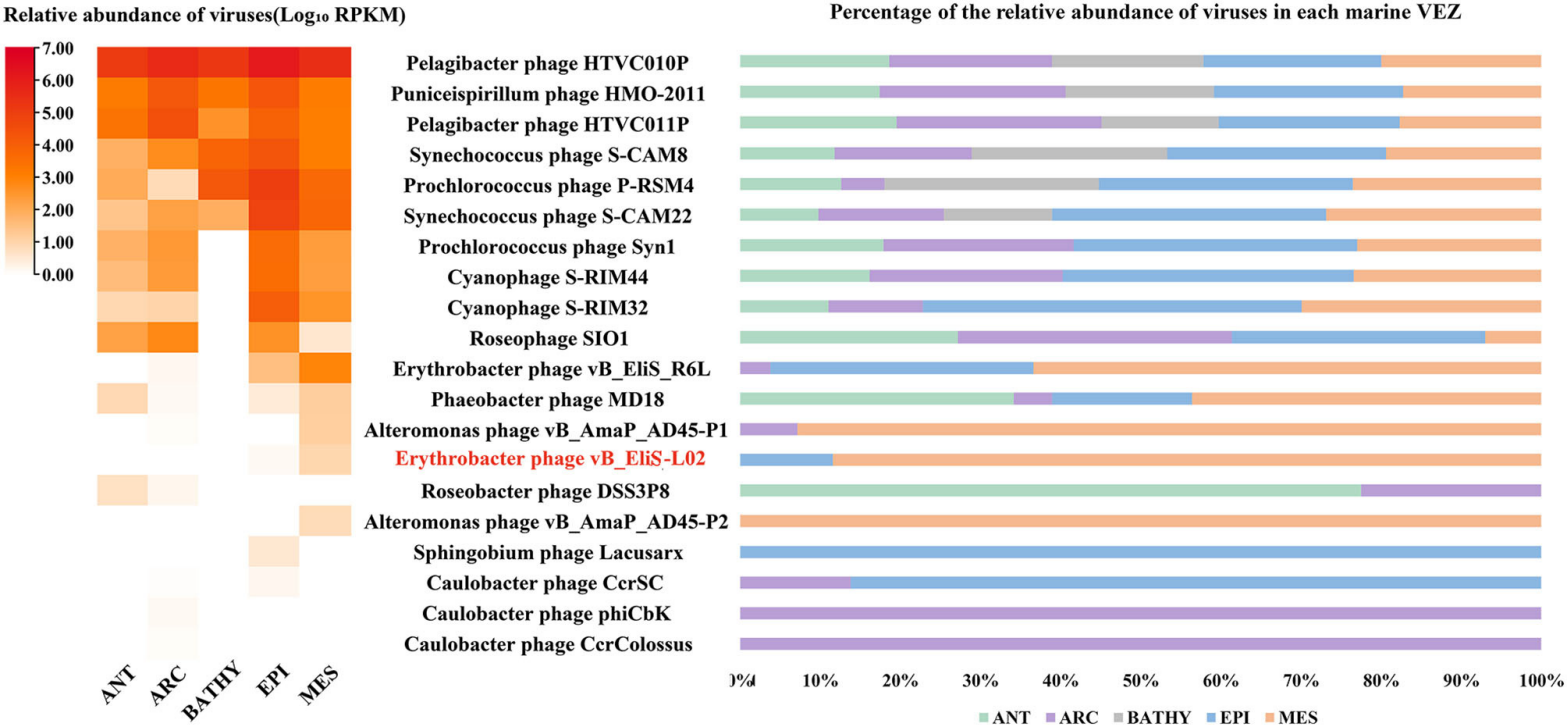
Relative abundance of Erythrobacter phage vB_EliS-L02 compared to the abundances of oceanic representative bacteriophages and Cbk-like phages. Relative abundances are expressed by RPKM (reads per kilobase per million mapped reads) values and described with log_10_ transformation. Left, relative abundances of different bacteriophages in different viral ecological zones (VEZs). Right, distribution patterns of bacteriophages in five VEZs with RPKM values normalized by the number of databases of each VEZ. ANT, Antarctic; ARC, Arctic; BATHY, bathypelagic; EPI, temperate and tropical epipelagic; MES, temperate and tropical mesopelagic.

Then, the genome of phage vB_EliS-L02 was mapped to the Global Ocean Viromes data set (GOV2.0), and five viral ecological zones (VEZs) were defined, including Arctic (ARC), Antarctic (ANT), bathypelagic (BATHY), temperate and tropical epipelagic (EPI), and temperate and tropical mesopelagic (MES), as per a previous study (Figure 6; Wang et al., 2021). The number of databases for each ecological environment was normalized before RPKM calculation. The results showed high abundances of Pelagibacter phage, the Puniceispirillum phage HMO-2011 that infects SAR116 clade, and Cyanophages in almost all VEZs, and Alteromonas phages were only abundant in MES, which were similar to some previous studies (Hurwitz and Sullivan, 2013; Kang et al., 2013; Zhao et al., 2013; Wang et al., 2021). All Cbk-like phages showed low abundance in detected VEZs, and vB_EliS-L02 were only detected in EPI and MES (Figure 6), indicating that Cbk-like phages had low abundance in the ocean.

## Conclusions

*Erythrobacter* is an important AAPB genus in the ocean, which may play an important role in the ocean carbon cycle. In this study, a novel Cbk-like Erythrobacter phage, vB_EliS-L02, was isolated and characterized. Phage vB_EliS-L02 genome contains a large number of tRNA and complex nucleotide biosynthetic and regulating systems, which help it to withstand adverse environmental conditions within the host. Phylogenetic and comparative genomic analysis suggested that phage vB_EliS-L02 was distinct from other isolated phages, and represented a new CbK-like siphoviral genus, named *Eliscbkvirus*. This study provided more information on the little-known Erythrobacter phage, and deepened our understanding of the phage–host interactions under complex environments.

## Data Availability Statement

The datasets presented in this study can be found in online repositories. The names of the repository/repositories and accession number(s) can be found at: https://www.ncbi.nlm.nih.gov/genbank/, OL955261.

## Author Contributions

XL, RG, and XZ performed the experiment and analyzed the data. YY collected water samples. LL designed the study. All authors listed assisted in writing the manuscript, discussed the results, and commented on the manuscript. All authors contributed to the article and approved the submitted version.

## Funding

This research was supported by the Shandong Provincial Natural Science Foundation (Grant No. ZR2021QC220).

## Conflict of Interest

YY and LL were employed by the Weihai Changqing Ocean Science Technology Co., Ltd. The remaining authors declare that the research was conducted in the absence of any commercial or financial relationships that could be construed as a potential conflict of interest.

## Publisher's Note

All claims expressed in this article are solely those of the authors and do not necessarily represent those of their affiliated organizations, or those of the publisher, the editors and the reviewers. Any product that may be evaluated in this article, or claim that may be made by its manufacturer, is not guaranteed or endorsed by the publisher.
