# Supplementary material for: The First Cbk-Like Phage Infecting Erythrobacter, Representing a Novel Siphoviral Genus
Source: Front Microbiol. 2022 May 10;13:861793. doi: 10.3389/fmicb.2022.861793 (PMC9127768; doi:10.3389/fmicb.2022.861793)
Supplement: Supplementary file 1 [file Data_Sheet_1.docx]

**SUPPLEMENTARY MATERIAL**

**Table S1** genome annotations of *Erythrobacter litoralis* DSM 8509 infecting phage vB_EliS-L02

| ORFs | Start | Stop | Strand | Putative function |
| --- | --- | --- | --- | --- |
| 1 | 90 | 302 | + | - |
| 2 | 786 | 1676 | + | PhoH Phosphate starvation-inducible protein PhoH, predicted ATPase |
| 3 | 1798 | 2340 | + | - |
| 4 | 2337 | 2873 | + | Hypothetical protein |
| 5 | 2873 | 3043 | + | - |
| 6 | 3466 | 5220 | + | portal protein |
| 7 | 5220 | 5390 | + | - |
| 8 | 5667 | 6455 | + | putative phage structural protein |
| 9 | 6598 | 7524 | + | putative major capsid protein |
| 10 | 7583 | 8305 | + | Hypothetical protein |
| 11 | 8339 | 8785 | + | putative minor capsid protein |
| 12 | 8867 | 9259 | + | - |
| 13 | 9475 | 9990 | + | virion structural protein |
| 14 | 9990 | 10373 | + | - |
| 15 | 10377 | 10871 | + | Hypothetical protein |
| 16 | 10868 | 11323 | + | Hypothetical protein |
| 17 | 11466 | 13265 | + | putative major tail tube protein |
| 18 | 13393 | 13797 | + | Hypothetical protein |
| 19 | 13875 | 14228 | + | Hypothetical protein |
| 20 | 14247 | 19055 | + | tail tape-measure protein |
| 21 | 19119 | 19754 | + | DUF2460 protein |
| 22 | 19751 | 21148 | + | F5/8 type C domain protein |
| 23 | 21225 | 21554 | + | phage cell wall peptidase NlpC/P60 family |
| 24 | 21554 | 25213 | + | phage tail protein |
| 25 | 25263 | 28262 | + | phage-related tail fiber protein |
| 26 | 28262 | 30868 | + | Putative phage tail protein |
| 27 | 30865 | 31560 | + | LamG domain-containing protein |
| 28 | 31569 | 32411 | + | 1,6-anhydro-N-acetylmuramyl-L-alanine amidase |
| 29 | 32423 | 32881 | + | Hypothetical protein |
| 30 | 32892 | 33080 | + | - |
| 31 | 33077 | 33946 | + | FAD-dependent thymidylate synthase |
| 32 | 34026 | 36173 | + | DNA ligase |
| 33 | 36177 | 36395 | + | - |
| 34 | 36392 | 37006 | + | 5'-nucleotidase |
| 35 | 37007 | 37828 | - | Transcriptional regulator, XRE family |
| 36 | 37973 | 38239 | - | ribonucleoside-diphosphate reductase subunit beta |
| 37 | 38241 | 39251 | - | ribonucleoside-diphosphate reductase subunit beta |
| 38 | 39251 | 39577 | - | Hypothetical protein |
| 39 | 39660 | 41426 | - | ribonucleoside-diphosphate reductase |
| 40 | 41542 | 42063 | - | putative RNA polymerase alpha subunit C-terminal domain protein |
| 41 | 42134 | 42436 | - | Hypothetical protein |
| 42 | 42759 | 42983 | - | - |
| 43 | 42980 | 43117 | - | - |
| 44 | 43114 | 43698 | - | Putative nucleotide pyrophosphohydrolase domain protein |
| 45 | 43707 | 44237 | - | - |
| 46 | 44267 | 44875 | - | putative deoxynucleotide monophosphate kinase |
| 47 | 44989 | 46326 | - | ATP-dependent RecD-like DNA helicase |
| 48 | 46357 | 46710 | - | - |
| 49 | 46786 | 47790 | - | Hypothetical protein |
| 50 | 47851 | 48210 | - | - |
| 51 | 48308 | 48601 | - | - |
| 52 | 48598 | 49659 | - | Hypothetical protein |
| 53 | 49659 | 50144 | - | - |
| 54 | 50141 | 50347 | - | Hypothetical protein |
| 55 | 50347 | 51027 | - | PolC-type DNA polymerase III |
| 56 | 51027 | 51383 | - | - |
| 57 | 51465 | 51554 | - | - |
| 58 | 51551 | 51778 | - | - |
| 59 | 51780 | 52349 | - | HYPO CcrColossus_gp190 |
| 60 | 52349 | 54631 | - | thermostable DNA polymerase I |
| 61 | 54774 | 54965 | + | - |
| 62 | 55144 | 55242 | - | - |
| 63 | 55286 | 55831 | - | Hypothetical protein |
| 64 | 55828 | 55926 | - | - |
| 65 | 55926 | 56261 | - | - |
| 66 | 56258 | 56425 | - | - |
| 67 | 56422 | 58071 | - | Hypothetical protein |
| 68 | 58074 | 58802 | - | Hypothetical protein |
| 69 | 58799 | 59275 | - | Cytidine and deoxycytidylate deaminase zinc-binding region（deoxycytidylate deaminase ） |
| 70 | 59275 | 59985 | - | Hypothetical protein |
| 71 | 60049 | 60234 | - | - |
| 72 | 60231 | 63053 | - | RNA polymerase-associated protein |
| 73 | 63066 | 64466 | - | transposase |
| 74 | 64466 | 66718 | - | Phage rIIA lysis inhibitor |
| 75 | 66717 | 67046 | + | Hypothetical protein |
| 76 | 67213 | 67593 | + | - |
| 77 | 67684 | 68310 | + | tyrosine recombinase |
| 78 | 68380 | 68862 | + | - |
| 79 | 69724 | 69876 | + | - |
| 80 | 70852 | 70950 | - | - |
| 81 | 71851 | 72162 | + | - |
| 82 | 75531 | 75644 | + | - |
| 83 | 76613 | 77062 | + | Proteasome, subunit alpha/beta |
| 84 | 77203 | 80226 | + | Hypothetical protein |
| 85 | 80306 | 81547 | + | RNA-splicing ligase |
| 86 | 81551 | 81796 | + | - |
| 87 | 81796 | 82227 | + | Hypothetical protein |
| 88 | 82227 | 82559 | + | - |
| 89 | 82642 | 82998 | + | Hypothetical protein |
| 90 | 82998 | 83477 | + | Hypothetical protein |
| 91 | 83598 | 84005 | + | - |
| 92 | 84046 | 84615 | + | putative protein metallo-phosphoesterase |
| 93 | 84623 | 84853 | + | - |
| 94 | 84934 | 85125 | - | - |
| 95 | 85122 | 85484 | - | Hypothetical protein |
| 96 | 85513 | 86196 | - | - |
| 97 | 86268 | 86528 | - | - |
| 98 | 86525 | 86788 | - | - |
| 99 | 86803 | 87750 | - | Hypothetical protein |
| 100 | 87855 | 88652 | - | Uncharacterized protein |
| 101 | 88723 | 89439 | - | - |
| 102 | 89441 | 90922 | - | putative nicotinate phosphoribosyltransferase |
| 103 | 90935 | 92011 | - | bifunctional NMN adenylyltransferase/nudix hydrolase |
| 104 | 92020 | 92424 | - | Hypothetical protein |
| 105 | 92421 | 92642 | - | - |
| 106 | 92658 | 92822 | - | - |
| 107 | 92819 | 93031 | - | - |
| 108 | 93044 | 93403 | - | DUF2958 domain-containing protein |
| 109 | 93462 | 93890 | - | Aminoacyl-tRNA hydrolase（peptidyl-tRNA hydrolase） |
| 110 | 93887 | 95824 | - | Hypothetical protein |
| 111 | 95967 | 96131 | - | - |
| 112 | 96133 | 96363 | - | - |
| 113 | 96505 | 96753 | - | - |
| 114 | 96840 | 97106 | - | - |
| 115 | 97103 | 97399 | - | Hypothetical protein |
| 116 | 97396 | 97653 | - | - |
| 117 | 97650 | 98939 | - | AAA family ATPase |
| 118 | 98941 | 99396 | - | Hypothetical protein |
| 119 | 99393 | 99869 | - | - |
| 120 | 99866 | 100219 | - | - |
| 121 | 100317 | 100508 | - | - |
| 122 | 100575 | 100739 | - | - |
| 123 | 100736 | 101146 | - | Hypothetical protein |
| 124 | 101143 | 101463 | - | - |
| 125 | 101463 | 101777 | - | - |
| 126 | 101837 | 102199 | - | - |
| 127 | 102199 | 102585 | - | Hypothetical protein |
| 128 | 102647 | 103039 | - | Hypothetical protein |
| 129 | 103039 | 103521 | - | Hypothetical protein |
| 130 | 103523 | 104500 | - | nucleotidyltransferase domain-containing protein |
| 131 | 104497 | 104721 | - | - |
| 132 | 104718 | 105059 | - | - |
| 133 | 105070 | 105348 | - | - |
| 134 | 105411 | 105728 | - | thioredoxin |
| 135 | 105725 | 106291 | - | Hypothetical protein |
| 136 | 106288 | 106656 | - | - |
| 137 | 106653 | 107369 | - | Hypothetical protein |
| 138 | 107414 | 108511 | - | - |
| 139 | 108508 | 109005 | - | Polynucleotide 5'-kinase and 3'-phosphatase （AAA family ATPase） |
| 140 | 109058 | 109348 | - | - |
| 141 | 109401 | 110084 | - | Hypothetical protein |
| 142 | 110081 | 110401 | - | - |
| 143 | 110543 | 110860 | - | - |
| 144 | 110875 | 111369 | - | Hypothetical protein |
| 145 | 111366 | 111662 | - | - |
| 146 | 111659 | 112102 | - | - |
| 147 | 112146 | 112412 | - | - |
| 148 | 112474 | 113004 | - | DUF1508 domain-containing protein |
| 149 | 113007 | 113168 | - | - |
| 150 | 113168 | 113296 | - | - |
| 151 | 113293 | 113547 | - | - |
| 152 | 113651 | 113947 | - | - |
| 153 | 114083 | 114319 | - | Hypothetical protein |
| 154 | 114316 | 114522 | - | - |
| 155 | 114522 | 114857 | - | - |
| 156 | 114985 | 115338 | - | - |
| 157 | 115338 | 115745 | - | Hypothetical protein |
| 158 | 115747 | 116478 | - | Hypothetical protein |
| 159 | 116607 | 116864 | - | - |
| 160 | 116925 | 117206 | - | - |
| 161 | 117270 | 117701 | - | - |
| 162 | 117701 | 118024 | - | Hypothetical protein |
| 163 | 118051 | 118524 | - | Hypothetical protein |
| 164 | 118524 | 118751 | - | - |
| 165 | 118748 | 118981 | - | - |
| 166 | 118987 | 119418 | - | Membrane protease subunit |
| 167 | 119565 | 119912 | - | - |
| 168 | 119909 | 120238 | - | - |
| 169 | 120238 | 120525 | - | Hypothetical protein |
| 170 | 120614 | 120940 | - | - |
| 171 | 120937 | 121422 | - | Hypothetical protein |
| 172 | 121422 | 121667 | - | - |
| 173 | 121667 | 121783 | - | - |
| 174 | 121867 | 122457 | - | Hypothetical protein |
| 175 | 122507 | 122773 | - | Hypothetical protein |
| 176 | 122804 | 122950 | + | - |
| 177 | 122985 | 124025 | - | Hypothetical protein |
| 178 | 124028 | 124357 | - | Hypothetical protein |
| 179 | 124429 | 124650 | - | - |
| 180 | 124713 | 124820 | - | - |
| 181 | 124817 | 125281 | - | - |
| 182 | 125278 | 125520 | - | - |
| 183 | 125517 | 125897 | - | MULTISPECIES: DUF1523 family protein |
| 184 | 126734 | 127090 | + | - |
| 185 | 127087 | 127422 | + | - |
| 186 | 127454 | 127735 | + | - |
| 187 | 127738 | 128106 | + | Hypothetical protein |
| 188 | 128115 | 128615 | + | crossover junction endodeoxyribonuclease |
| 189 | 128619 | 129173 | + | putative terminase small subunit |
| 190 | 129170 | 130927 | + | terminase-like family protein |
| 191 | 131142 | 131351 | + | Hypothetical protein |
| 192 | 132127 | 132276 | + | - |
| 193 | 132609 | 132917 | + | - |
| 194 | 133136 | 133564 | + | - |
| 195 | 133718 | 133885 | + | - |
| 196 | 133987 | 134250 | + | - |
| 197 | 134397 | 134705 | + | - |
| 198 | 134719 | 134925 | + | Hypothetical protein |
| 199 | 135044 | 135214 | + | - |
| 200 | 135243 | 135389 | + | - |
| 201 | 135549 | 135800 | + | Hypothetical protein |
| 202 | 135797 | 135997 | + | Hypothetical protein |
| 203 | 136152 | 136349 | + | Hypothetical protein |
| 204 | 136346 | 136588 | + | - |
| 205 | 136744 | 137070 | + | Hypothetical protein |
| 206 | 137304 | 137696 | + | - |
| 207 | 137856 | 138140 | + | Hypothetical protein |
| 208 | 138302 | 138694 | + | - |
| 209 | 138853 | 139344 | + | Hypothetical protein |
| 210 | 139490 | 139861 | + | Hypothetical protein |
| 211 | 140007 | 140453 | + | Hypothetical protein |
| 212 | 140655 | 141008 | + | Hypothetical protein |
| 213 | 141153 | 141947 | + | Hypothetical protein |
| 214 | 142101 | 142679 | + | - |
| 215 | 142863 | 142991 | + | - |
| 216 | 142988 | 143677 | + | Hypothetical protein |
| 217 | 143781 | 143951 | + | - |
| 218 | 144223 | 144486 | + | - |
| 219 | 144634 | 145203 | + | - |
| 220 | 145405 | 145689 | + | Hypothetical protein |
| 221 | 145686 | 145934 | + | - |
| 222 | 146523 | 146855 | + | Hypothetical protein |
| 223 | 147049 | 147261 | + | - |
| 224 | 147620 | 147877 | + | - |
| 225 | 148108 | 148314 | + | - |
| 226 | 148314 | 148508 | + | Hypothetical protein |
| 227 | 148570 | 148779 | + | - |
| 228 | 149026 | 149202 | + | - |
| 229 | 149217 | 149486 | + | - |
| 230 | 149662 | 149820 | + | - |
| 231 | 149817 | 149969 | + | - |

**Table S2** Genomic annotation of conserved domains detected in phage vB_EliS-L02 genome

| ORF | Start | Stop | Strand | Function | CD accession no. | Matched phage | E vaule |
| --- | --- | --- | --- | --- | --- | --- | --- |
| **Phage structure** | | | | | | | |
| 8 | 5667 | 6455 | + | Phage structural protein | YP_009600736 | Sphingobium phage Lacusarx | 1.75E-50 |
| 9 | 6598 | 7524 | + | Major capsid protein | YP_009600737 | Sphingobium phage Lacusarx | 5.01E-136 |
| 11 | 8339 | 8785 | + | Minor capsid protein | YP_009600738 | Sphingobium phage Lacusarx | 2.14E-31 |
| 13 | 9475 | 9990 | + | Virion structural protein | YP_009600740 | Sphingobium phage Lacusarx | 1.44E-43 |
| 17 | 11466 | 13265 | + | Major tail tube protein | YP_009600744 | Sphingobium phage Lacusarx | 2.14E-200 |
| 24 | 21554 | 25213 | + | Phage tail protein | WP_063185755 | Chelatococcus daeguensis | 6.51E-123 |
| 25 | 25263 | 28262 | + | Tail fiber protein | WP_140041953 | Sphingobium fuliginis | 4.63E-47 |
| 26 | 28262 | 30868 | + | Phage tail protein | YP_224012 | Alphaproteobacteria virus phiJl001 | 8.44E-50 |
| **Phage packing** | | | | | | | |
| 6 | 3466 | 5220 | + | Portal protein | YP_009600734 | Sphingobium phage Lacusarx | 3.75E-218 |
| 20 | 14247 | 19055 | + | Tail tape measure protein | YP_009600748 | Sphingobium phage Lacusarx | 6.72E-248 |
| 189 | 128619 | 129173 | + | Terminase small subunit | YP_009810167 | Caulobacter phage CcrBL9 | 3.48E-05 |
| 190 | 129170 | 130927 | + | Terminase large subunit | YP_009600900 | Sphingobium phage Lacusarx | 9.24E-225 |
| **Host lysis** | | | | | | | |
| 22 | 19751 | 21148 | + | F5/8 type C domain protein | YP_009600750 | Sphingobium phage Lacusarx | 6.94E-105 |
| 23 | 21225 | 21554 | + | NlpC/P60 family | YP_009600751 | Sphingobium phage Lacusarx | 1.35E-17 |
| 27 | 30865 | 31560 | + | LamG domain-containing protein | RYE41603.1 | Hyphomicrobiales bacterium | 1.00E-27 |
| 28 | 31569 | 32411 | + | 1,6-anhydro-N-acetylmuramyl-L-alanine amidase | YP_009600755 | Sphingobium phage Lacusarx | 1.41E-67 |
| 74 | 64466 | 66718 | - | rIIA lysis inhibitor | YP_009600805 | Sphingobium phage Lacusarx | 1.43E-159 |
| **DNA replication, recombination and repair functions** | | | | | | | |
| 32 | 34026 | 36173 | + | DNA ligase | YP_009600758 | Sphingobium phage Lacusarx | 1.33E-199 |
| 35 | 37007 | 37828 | - | HTH-type transcriptional regulator | YP_009600761 | Desulfotomaculum reducens MI-1 | 1.96E-02 |
| 36 | 37973 | 38239 | - | ribonucleoside-diphosphate reductase subunit beta | YP_009600762 | Sphingobium phage Lacusarx | 3.32E-16 |
| 37 | 38241 | 39251 | - | ribonucleoside-diphosphate reductase subunit beta | YP_009600762 | Sphingobium phage Lacusarx | 1.46E-110 |
| 39 | 39660 | 41426 | - | ribonucleoside-diphosphate reductase subunit alpha | YP_009600764 | Sphingobium phage Lacusarx | 2.82E-205 |
| 40 | 41542 | 42063 | - | RNA polymerase alpha subunit C-terminal domain protein | WP_138392197 | Rhizobium sp. MHM7A | 7.61E-21 |
| 47 | 44989 | 46326 | - | ATP-dependent RecD-like DNA helicase | YP_009600778 | Sphingobium phage Lacusarx | 8.58E-71 |
| 55 | 50347 | 51027 | - | PolC-type DNA polymerase III | YP_009600786 | Sphingobium phage Lacusarx | 1.05E-62 |
| 60 | 52349 | 54631 | - | Thermostable DNA polymerase I | YP_009600788 | Sphingobium phage Lacusarx | 2.24E-285 |
| 72 | 60231 | 63053 | - | RNA polymerase-associated protein | YP_009600800 | Sphingobium phage Lacusarx | 3.29E-188 |
| 73 | 63066 | 64466 | - | transposase | YP_009600804 | Sphingobium phage Lacusarx | 2.92E-103 |
| 77 | 67684 | 68310 | + | tyrosine recombinase | YP_009600809 | Sphingobium phage Lacusarx | 5.75E-40 |
| 85 | 80306 | 81547 | + | RNA-splicing ligase | YP_009600818 | Sphingobium phage Lacusarx | 9.48E-156 |
| 117 | 97650 | 98939 | - | AAA family ATPase | WP_135282878 | Candidatus Macondimonas diazotrophica | 6.41E-78 |
| 139 | 108508 | 109005 | - | AAA family ATPase | MK817115 | Escherichia phage vB_EcoM_phAPEC6 | 1.31E-04 |
| 188 | 128115 | 128615 | + | Crossover junction endodeoxyribonuclease | YP_009600899 | Sphingobium phage Lacusarx | 6.36E-47 |
| **AMGs** | | | | | | | |
| 2 | 786 | 1676 | + | PhoH | LR798231 | uncultured Caudovirales phage | 1.75E-50 |
| 31 | 33077 | 33946 | + | FAD-dependent thymidylate synthase | YP_009600772 | Sphingobium phage Lacusarx | 4.64E-85 |
| 34 | 36392 | 37006 | + | 5'-nucleotidase |  | Sphingobium phage Lacusarx | 7.94E-15 |
| 44 | 43114 | 43698 | - | Putative nucleotide pyrophosphohydrolase domain protein | MN820898 | Sphingomonas phage vB_StuS_MMDA13 | 3.48E-32 |
| 46 | 44267 | 44875 | - | Deoxynucleotide monophosphate kinase | YP_009600773 | Sphingobium phage Lacusarx | 1.52E-55 |
| 69 | 58799 | 59275 | - | Cytidine and deoxycytidylate deaminase zinc-binding region（deoxycytidylate deaminase ） | VVB53280.1 | uncultured archaeon | 1.00E-44 |
| 83 | 76613 | 77062 | + | Proteasome, subunit alpha/beta | LR798198 | uncultured Caudovirales phage | 2.37E-14 |
| 92 | 84046 | 84615 | + | putative protein metallophosphoesterase | QGH74700 | Bacteriophage DSS3_PM1 | 1.00E-19 |
| 102 | 89441 | 90922 | - | putative nicotinate phosphoribosyltransferase | YP_009600839 | Sphingobium phage Lacusarx | 7.43E-177 |
| 103 | 90935 | 92011 | - | bifunctional NMN adenylyltransferase/nudix hydrolase | YP_009600840 | Sphingobium phage Lacusarx | 1.81E-93 |
| 109 | 93462 | 93890 | - | peptidyl-tRNA hydrolase | QGH74801.1 | Bacteriophage DSS3_MAL1 | 2.00E-16 |
| 130 | 103523 | 104500 | - | nucleotidyltransferase domain-containing protein | WP_184246834 | Novosphingobium chloroacetimidivorans | 2.88E-84 |
| 134 | 105411 | 105728 | - | thioredoxin | WP_152709470 | Microvirga tunisiensis | 2.96E-12 |
| 166 | 118987 | 119418 | - | Membrane protease subunit | RYY37440 | Sphingomonadales bacterium | 2.00E-61 |
| **Unknown function** | | | | | | | |
| 21 | 19119 | 19754 | + | DUF2460 protein | YP_009600749 | Sphingobium phage Lacusarx | 1.64E-61 |
| 108 | 93044 | 93403 | - | DUF2958 domain-containing protein | WP_130690265 | Rhizobium leguminosarum | 1.60E-26 |
| 148 | 112474 | 113004 | - | DUF1508 domain-containing protein | WP_138616601 | Qipengyuania marisflavi | 5.20E-17 |
